# Exploration of Polyphenols Extracted from *Cytisus* Plants and Their Potential Applications: A Review

**DOI:** 10.3390/antiox13020192

**Published:** 2024-02-02

**Authors:** Diana Ferreira-Sousa, Zlatina Genisheva, María Jesús Rodríguez-Yoldi, Beatriz Gullón, Carlos E. Costa, José A. Teixeira, Cláudia M. Botelho, Pedro Ferreira-Santos

**Affiliations:** 1 CEB—Centre of Biological Engineering, University of Minho, Campus de Gualtar, 4710-057 Braga, Portugal; id10389@alunos.uminho.pt (D.F.-S.); carlos.costa@ceb.uminho.pt (C.E.C.); jateixeira@deb.uminho.pt (J.A.T.); 2 CVR—Centre of Wastes Valorization, 4800-058 Guimarães, Portugal; zlatina@cvresiduos.pt; 3 Pharmacology and Physiology and Legal and Forensic Medicine Department, Veterinary Faculty, Zaragoza University, 50009 Zaragoza, Spain; mjrodyol@unizar.es; 4 CIBERobn, ISCIII, IIS Aragón, IA2, 50009 Zaragoza, Spain; 5 Department of Chemical Engineering, Faculty of Science, University of Vigo, 32004 Ourense, Spain; bgullon@uvigo.es; 6 IAA—Instituto de Agroecoloxía e Alimentación, University of Vigo (Campus Auga), 32004 Ourense, Spain; 7 LABBELS—Associate Laboratory, Braga/Guimarães, 4710-057 Braga, Portugal

**Keywords:** bioactive compounds, *Cytisus* by-products, food applications, green valorization, innovative extraction, therapeutic actions

## Abstract

The increasing world population means an increased demand for sustainable processes and products related to foods, particularly those with added health benefits. Plants can be an alternative source of nutritional and biofunctional ingredients. *Cytisus* plants are an underexploited bioresource, currently prevalent in the Mediterranean Basin and western Asia. This manuscript addresses the processing potential of *Cytisus* plants for the development of added-value products, including food formulations, food packaging, cosmetics, and therapeutic applications. Most research has reported that *Cytisus* spp. are a promising source of inexpensive bioactive polyphenol compounds. *Cytisus* flowers should be considered and exploited as raw materials for the development of new food ingredients (antioxidants, preservatives, additives, etc.), nutraceuticals, or even direct therapeutic agents (anticancer, antibacterial, etc.). In order to evaluate the socioeconomic effect of these underutilized plants, more research is needed to assess their valorization for therapeutic and dietary possibilities, as well as the economic impact.

## 1. Introduction

As the global population increases (by 2050, the world population may reach 9–10 billion), higher demand for more feedstock for food, pharmaceuticals, energetic alternatives, and attractive bioproducts is expected. Therefore, it is critical that industrial processes and resulting products grow according to sustainable practices relative to global demand [1,2]. 

Nowadays, the handling and processing of underexploited plants by-products and waste do not assure efficient use of these low-cost bioresources, even though some minor uses, such as biomass production and animal feed, has been reported. Plant by-products and waste can offer innovative possibilities for the development of added-value products and a reduction in their environmental impact [3]. It is indispensable to further reinforce the combination of suitable strategies to reduce, reprocess, and recycle organic agri-food biomass. These strategies will lead to their integral use and obtention of high-value ingredients, generating a minimal part of untreated and underused agro-industrial biomass, which can be discarded through incineration or uncontrolled landfill [4]. The reprocessing of plant biomass allows for added value of this bioresources and supports the bioeconomy. Plants and their by-products (e.g., bark, peels, pomace, seeds, flowers, etc.) are a potential source of nutritional and non-nutritional compounds. Among other constituents, the ones with the highest biofunctional and nutritional value should be highlighted, particularly primary metabolites including dietary fibers, proteins, and polysaccharides and bioactive secondary metabolites, i.e., phytochemicals like polyphenolics, carotenoids, alkaloids, and terpenes [5].

**Figure 1:**
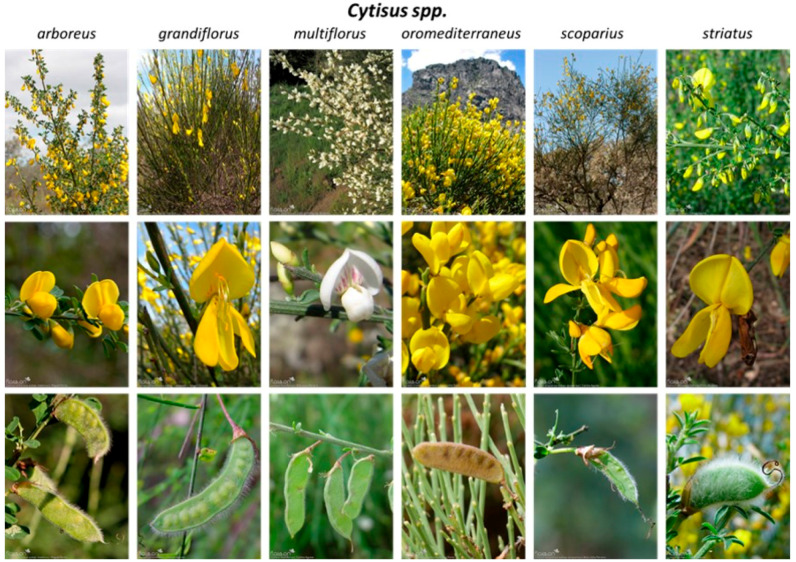
*Cytisus* species most prevalent in the Mediterranean basin region [14].

Plants of the genus *Cytisus*, better known as brooms, are the focus of this review article. The genus *Cytisus* includes about 70 species belonging to the family Fabaceae. Brooms are flowering shrubs, subshrubs, or small trees with alternate branches and unifoliate to trifoliate leaves, deciduous to evergreen. Flowers are single or paired up to four leaf axils, usually with a two-lipped, top lip minutely toothed calyx. The fruits are mostly typical pods with more or less explosive (and valvular) dehiscence via dorsal and ventral sutures (Figure 1) [6]. These plants are distributed all over the world, being prevalent to open sites (typically scrub and heathland) of the Mediterranean Basin (Europe and North Africa) and western Asia [7].

*Cytisus* plants are of agricultural interest, allowing nitrogen fixation in soils due to *Rhizobium* nodules on roots. However, in recent years, they are starting to present a problem for vegetation in many countries, and are considered weeds. It has been noted that these species have great expression worldwide due to their rapid growth, creating dense stands. This evidence can lead to problems such as inaccessibility for wildlife and the risk of fires [6]. Thus, in some countries, *Cytisus* plants are undervalued and considered a threat to other species; however, they can be an interesting source of valuable compounds [8,9]. In addition, plants of this genus have been traditionally used for infusions and decoctions, due to their significant number of therapeutic properties [10,11,12]. These properties seem to be related to their rich content of bioactive constituents, like polyphenolic compounds [9,13].

As mentioned before, the need for food products with a “clean label”, as well as nutraceuticals and natural drugs (pharmaceuticals), motivated by important factors such as health and sustainability, are the cause of the boom in research in this field. The consumer’s interest in natural, functional, and health-promoting products that are plant-based and obtained through environmentally friendly processes (green technologies) is pressuring this area of research. Therefore, the valorization of underexplored plants and the reuse of agricultural residues, in this case *Cytisus* plants, may be a viable option to develop products of interest for the food and pharmaceutical industries, such as antioxidants, dyes, flavorings, antimicrobial agents, or isolated drugs with highlighted therapeutic potential.

This review is dedicated to generating comprehensive information for sustainable valorization of the underexploited *Cytisus* spp. plants. Hence, this manuscript addresses different species of *Cytisus* plants and their sustainable processing for a green recovery of bioactive polyphenols, as well as the development of valuable products containing these compounds, that can be used worldwide for food and therapeutic applications.

## 2. Recovery Strategies of Bioactive Polyphenols

The recovery of natural molecules (bioactives, pigments, proteins, biopolymers, etc.) is an imperative step to enable the (re)valorization of plant bioresources for consequent applications in food enrichment and preservatives, nutraceuticals, pharmaceutical, and cosmetic products.

Specifically, the effective recovery of bioactive molecules, e.g., polyphenols from natural sources, is highly influenced by the extraction technology applied. These methods must meet a number of necessities such as versatility, cost-effectiveness, environmental friendliness, and ease of operation, as well as guarantee high extraction yields and maintain the quality of the extracted phytochemicals [15].

Bioactive molecules are normally obtained using solid–liquid extraction (SLE). The efficiency of the extraction processes depends on several aspects related to the procedure used, like temperature, time, pH, and solvent, as well as raw materials composition [16]. Conventional approaches, such as Soxhlet extraction, hydrodistillation, and maceration, among others, are inarguably the most used for the recovery of valuable compounds [17]. These methods do have certain drawbacks, such as the destruction of thermolabile compounds caused by high temperatures maintained throughout the prolonged extraction period, limited target component recovery, low product quality, toxicological effects of resulting bioproducts, safety hazards due to a large amount of organic solvents used (harmful to the environment and human health), and high energy consumption (high energy inputs required for distillation) [18,19]. Therefore, there is the need to develop and optimize comprehensive methodologies (mainly using green solvents and technologies) to improve bioactive phenolic compound (and others) extraction, particularly from plants and vegetal biomass, where the cell wall can alter/impair the extraction efficiency.

New sustainable extraction techniques have been developed in an effort to address these problems and increase the quality and performance of the extraction process. When compared to conventional ones, green technologies have some advantages, including quicker heating and higher thermal efficiency, shorter extraction time, less solvent usage, and high selectivity [19,20]. In general, another important feature of these green technologies is their energy-saving efficiency (eco-friendly).

**Figure 2:**
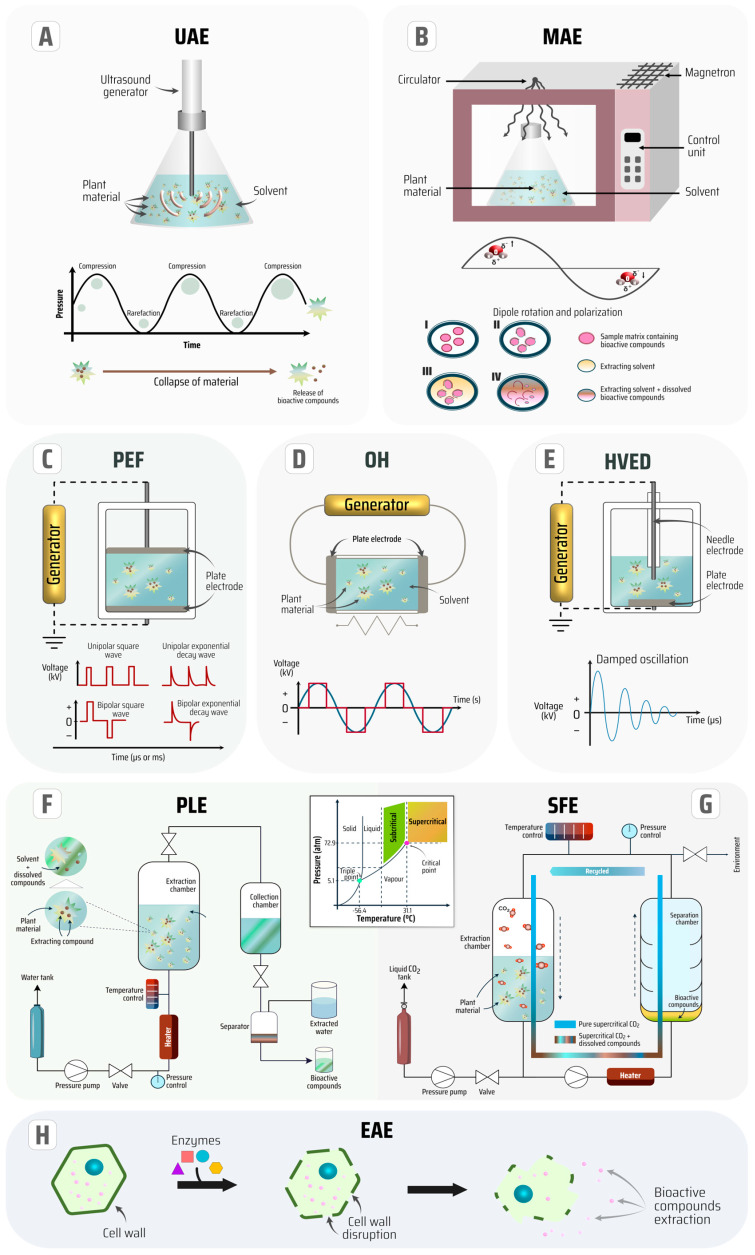
Illustration of innovative extraction technologies of bioactive compounds (**A**–**H**).

Thus, for the isolation of phenolic compounds, alternative green extraction technologies such as ultrasound-assisted extraction (UAE), microwave-assisted extraction (MAE), electrotechnologies-assisted extraction like pulsed-electric fields (PEF), high voltage electrical discharges (HVED) and ohmic heating (OH), pressurized liquid extraction (PLE), supercritical fluid extraction (SFE), and enzyme-assisted extraction (EAE) have been successfully applied for several bioresources [4,18,21,22]. These technologies are illustrated in Figure 2 to elucidate the extraction processes of bioactive phytochemicals from plant material. Furthermore, the concept of each innovative technique for the sustainable extraction process, as well as some advantages and disadvantages contrasting each of them, are summarized in Table 1.

The next paragraphs address a few studies reported in the literature regarding the extraction of phenolic compounds from several *Cytisus* species. While using conventional extraction, numerous types of solvents (water or organic) have been tested for the effective extraction of these compounds from *Cytisus* biomass. Solvents such as ethanol, methanol, acetone, diethyl ether, isopropanol, ethyl acetate, chloroform, and ethyl lactate, as well as their combinations with water, have been the most used. For example, *C. scoparius* branches were previously treated with hexane to remove hydrophobic components in a Soxhlet extractor. The extracted solid was first processed with an acetone:water mixture (40 °C for 1 h) and its raffinate was extracted with acidified 70% ethanol (55 °C for 1 h) in an orbital shaker. The authors indicated that the extraction yield was 10 times higher in the acetone/water extracts than in the acidified ethanol extracts [23]. Sundararajan et al. [24] also used conventional SLE with ethanol:water (7:3 ratio) for the recovery of antioxidant compounds from *C. scoparius* aerial parts. Their results showed that the powdered extracts obtained from *C. scoparius* have a higher antioxidant capacity than the standard compounds used.

Barros et al. [25] determined the phenolic recovery and profiles of several plants used for traditional or folk medicine, including *C. multiflorus*. The results show that hydromethanolic extraction (80% methanol) was effective in extracting phenolic compounds, mainly flavonoids (54.5 mg/g plant). Recently, Larit et al. [26] has used hydroethanolic solvent (80% ethanol) for selective extraction of phenolic compounds from Algerian plant *Cytisus villosus* Pourr. The authors indicated that n-butanol fractioned ethanolic extract from *C. villosus* aerial parts had a higher total phenolic content (363 mg GAE/g extract) and antioxidant activity than the ethyl acetate (208 mg GAE/g extract) and chloroform (56 mg GAE/g extract) fractioned extracts.

Lores et al. [8] considered the viability of using ethyl lactate (an environmentally friendly solvent) to obtain solubilized phenolic compounds from different parts (flowers, seeds, pods, and branches) of *C. scoparius*, and evaluated the characteristics obtained extracts by PLE (120 °C; 10 min; hydro-organic mixtures of ethyl lactate) with those obtained with methanol (hazardous solvent). The results indicated that the PLE process was significantly better than the ambient-temperature columns, but it also consumed significantly more energy. In addition, a mixture of water (35%) and ethyl lactate (65%) proved to be a successful solvent for extracting *C. scoparius* polyphenols, resulting in extracts with high concentrations of natural polyphenols with great antioxidant activity (more than 35 mg GAE/g and around 4 mM Trolox/g of whole *C. scoparius* parts) comparatively to PLE of 100% ethyl lactate (35 mg GAE/g and 2 mM Trolox/g of whole *C. scoparius* parts) and conventional extraction with methanol (32 mg GAE/g and around 3.8 mM Trolox/g of whole *C. scoparius* parts).

González et al. [23] compared different extraction technologies to recover phenolics from branches of *C. scoparius*: conventional solvent extraction using ethanol, and extraction with PLE (water) or with SFE (SC-CO_2_). The authors found that PLE autohydrolysis can be a suitable technique with respect to conventional solvent methods in order to increase extraction yields. In addition, the stage by SC-CO_2_ extraction allows obtaining a raffinate with similar free radical scavenging properties to the ethanolic extract. Interestingly, microwave hydro-diffusion and gravity (MHG) may be an emergent solvent-free alternative to obtain selective phytochemicals in a sustainable manner [7]. In this context, López-Hortas et al. [7] evaluated the potential of MHG technology for recovering high-value-added compounds from *C. scoparius* flowers for sun cream applications. The results prove that the MHG extract has a higher content in lipids and carotenoids compared to the ethanolic extract obtained by SLE. On the other hand, this technology does not seem to be the most suitable for extracting phenolic compounds, proteins, and sugars from bioresources.

In the specific case of this review on *Cytisus* plants, as far as we know, in contrast to traditional SLE methods, only PLE, SFE, and MHG are used as alternative methodologies for recovering phenolic compounds. However, these methodologies must be optimized to maximize extraction yield, reduce the negative impact on target compounds, and increase energy efficiency. In this sense, more sustainable alternatives (other innovative technologies mentioned above), and their combination with alternative and non-harmful solvents (such as natural deep eutectic solvents (NADES)) should be explored to increase the application value of *Cytisus* spp. to obtain high-value products. These alternatives, if optimized, often involve the more sustainable valorization of renewable feedstocks with lower toxic solvents and reduced environmental impact, meeting the principles of Green Chemistry.

**Table 1:** Concept, advantages, and drawbacks of innovative techniques for sustainable recovery of bioactive phytochemicals from plants.

Extraction Technique	Concept	Advantages (vs. Conventional Methods)	Drawbacks	Reference
**Ultrasound-assisted extraction ** **(UAE)**	- Sound wave between 20 kHz and 100 MHz - Promote cavitation effects and cell wall disruption - Release of intracellular phenolic compounds	- Reduced extraction time - Low solvent use - Increased mass transfer - Temperature control - Preservation of heat-sensitive compounds - Ecological and inexpensive	- Invasive process for the operator - Intensive labor and attention - Limited extraction efficiency - Filtration required - Difficult to scale-up	[27,28]
**Microwave-assisted extraction ** **(MAE)**	- Electromagnetic radiation (frequencies 300 MHz to 300 GHz) - Diffusion of solvent into the biomass through cell pores and rupture of membranes - Release of intracellular phenolic compounds	- Rapid, selective, and uniform heating - Low organic solvent use - High effectiveness - Accelerated extraction process - Wide range of applications	- Chemical modifications of phenolic compounds by high temperature and pressure applications - Clean-up mandatory - Filtration required - Relatively expensive technique	[29,30]
**Pulsed electric field ** **(PEF)**	- Request pulses from 100–300 V/cm to 20–80 kV/cm - Stimulation of electroporation phenomenon - Release of intracellular phenolic compounds	- Non-thermal technology - Increase cell permeability - Preservation of heat-sensitive compounds - Selective extraction - Low organic solvent use - Accelerated extraction process - Inactivation of microorganisms	- Cell membranes can be reversible or irreversible during the electroporation mechanism - High equipment cost - Requires a significant amount of electrical energy - Limited application range (matrix dependent)	[4,31,32,33]
**Ohmic heating ** **(OH)**	- Non-pulsed electrotechnology - Conversion of electric fields into thermal energy - Applied voltage 400 and 4000 V (electric field from 0.001 to 1 kV/cm) - Induce the electroporation of the plant cell walls and membranes - Release of intracellular phenolic compounds	- Fast and uniform heating - Less energy consumption - Low organic solvents use - Decrease waste generation - Selective extraction - Accelerated extraction process - Inactivation of microorganisms	- Thermal impact on phenolic compounds - Limited by the viscosity and electrical conductivity of solvents and plant biomass - Reversible or irreversible electroporation mechanism - Requires more studies for scale-up	[4]
**High-voltage electrical discharges ** **(HVED)**	- Application of electrohydraulic discharge (20–80 kV/cm) - Generation of bubbles, UV radiations, and active radicals - Cell tissue fragmentation and destruction - Improve the mass transfer of phenolics for the solvent	- Accelerated extraction process - Low organic solvent use - Non-thermal technology - Inactivation of microorganisms	- Oxidation process and free radicals’ formation (can react with phenolic compounds) - Low selectivity	[34,35]
**Pressurized liquid extraction** **(PLE)**	- Liquid solvents at temperature/pressure above the atmospheric boiling point and below the critical point - Decrease solvent viscosity - Improve dissolution kinetics - Disruption of biomass structure - Solubilization of phenolic compounds	- Accelerated extraction process - Low organic solvent use - Versatility	- Expensive equipment - Clean-up required - Possible degradation of thermolabile phenolic compounds	[35,36]
**Supercritical fluid extraction** **(SFE)**	- Transformation of gas (CO_2_) into a supercritical fluid by application of temperature and pressure - Co-solvent (e.g., ethanol) used for phenolic compounds extraction	- Accelerated extraction process - Selective extraction (non-polar, mid-polar, or polar compounds) - No residual solvents - Non-toxicity, non-flammability of CO_2_	- Expensive technique - Matrix dependent - High energy consumption - Equipment complexity	[36,37]
**Enzyme-assisted extraction** **(EAE)**	- Enzymatic catalysis by specific and selective reactions - Enzymes to break down cell walls and facilitate the release of target compounds - Enzymes play a key role in hydrolyzing structural components	- High extraction efficiency - No organic solvents use	- Expensive enzyme costs - Specific environmental factors (temperature, pH, etc.) - Time-consuming	[36,38]

## 3. Phytochemical Composition of *Cytisus* Extracts

*Cytisus* spp. contains more than 70 phytoconstituents belonging to different groups. It is reported that the stem extracts of *C. multiflorus* species are very rich in alkaloids, namely sparteine, with anti-arrhythmic properties. Alkaloids have been shown to provide the therapeutic effect of many plant materials; however, most of the alkaloids are toxic [39]. Over 200 alkaloids have been identified in 300 plant species of up to 13 families [40]. Alkaloids are the major chemical compound found in *C. scoparius*, specifically quinolizidine [41] and lupanine [8]. Other compounds were also identified in the extracts of *C. scoparius*, including alkenols, benzenoids, phenylpropanoids, carotenoids, alkanones, steroids, lipids, monoterpenes, and polyphenols [42]. The essential oils of *C. triflorus* L’Her are rich in monoterpenes and sesquiterpenes, especially in the flowering stage of the plant. The other identified compounds belong to the groups of hydrocarbons, ketones, alcohols, esters aldehydes, fatty acids, and polyphenols. The major oils components are *b*-linalool, geraniol, retinal, *p*-mentha-1,4-dien-8-ol, γ-terpinene, and eugenol [10].

Plant extracts are rich in phytochemical constituents, though their medicinal properties and bioactivities are a result of the synergetic effect of all the present molecules. Plant extracts are commonly rich in polyphenol compounds belonging to different groups, playing vital roles in human health. On the basis of their chemical structure, phenolic compounds can be divided into two major groups: flavonoid (including flavanols, flavonols, flavones, flavanones, anthocyanins, isoflavones, and tannins) and non-flavonoid (including phenolic acids like hydroxybenzoic acids and hydroxycinnamic acids, stilbenes, and lignans) [33]. For instance, polyphenol compounds are present in a small amount in nature, and display important actions in biofunctionalized products. Moreover, current research reports that part of the functional activity of polysaccharides and proteins is closely related to the bound polyphenolic compounds, that is, to the presence of molecular complexes [43]. Polyphenols are known to have diverse biological properties between them, including antioxidant, anti-inflammatory, anticancer, and many others, and appear to be essential in assisting the body’s redox balance (homeostasis).

The *Cytisus* spp. are used in folk medicine as diuretics, hypnotics, sedatives, anti-diabetics, and hepatoprotectives, among other purposes [40]. According to Luís et al. [40] ethanolic extract of *Cytisus* spp. Has more polyphenols than that of the aqueous extract. For the extracts of *C. multiflorus*, high content of polyphenol compounds has been reported: 142.4 mg GAE/g dry matter, 176.1 mg GAE/g dry matter, 120.4 mg GAE/g dry matter, and 155.1 mg GAE/g dry matter for stems, leaves, flowers, and fruits, respectively. Leaves, stems, flowers, and fruit extracts were analyzed for their individual polyphenol compounds and antioxidant activity. Leaf extracts were the richest in terms of concentration of identified polyphenolic compounds, with the highest concentrations observed for ellagic acid (16.1 mg/g dry matter). The most representative compound in the extracts of fruit and flowers was ferulic acid, at 25.4 mg/g and 13.0 mg/g dry matter, respectively. The stem extracts were richest in quercetin (37.1 mg/g dry matter). The antioxidant properties of the *C. multiflorus* extracts were considered to have strong to moderate activity.

Extracts from different parts of *C. scoparius* were evaluated for their polyphenol profile [8]. The most abundant polyphenols found in the extracts of the hole plant were: rutin (110 μg/g dw), isoquercetin (62.7 μg/g dw), chrysin (119 μg/g dw), and quercetin (70 μg/g dw). The compound orientin was specifically found in extracts from pods (220 μg/g dw). Nevertheless, kaempferol (309 μg/g dw) was the most abundant polyphenol in pod extracts, but was not found in the whole plant extracts. Rutin (562 μg/g dw) is mostly found in the extracts of flowers, together with quercetin (210 μg/g dw) and isoquercetin (242 μg/g dw). The flower extracts were the richest extracts in polyphenols, while the branch extracts showed low concentrations of polyphenols. All extracts demonstrated high levels of antioxidant activity. The antibacterial activity was dependent on the type of the solvent used. Moreover, antibacterial activity was registered only for Gram-positive bacteria. Sundararajan et al. [24] also studied the antioxidant activity of hydro-alcoholic extracts of the areal parts of *C. scoparius*, using different evaluation methods. The results demonstrated that these extracts are a potential source of natural antioxidants. The antimicrobial activities of an ethyl acetate extract from *C. triflorus* L’Her were also evaluated [44]. Once again, the extracts expressed antibacterial activity only against Gram-positive bacteria. Moreover, the extracts were rich in chrysin derivatives and had strong antioxidant activity.

The ethyl acetate and aqueous extracts obtained from the aerial parts of *C. villosus* Pourr were studied for their chemical composition, antioxidant, antimicrobial, and antiproliferative activities [9]. Both types of extracts had rich polyphenol composition. Epigallocatechin (111 μg/g dw) was the major polyphenol compound found in the aqueous extracts, and myricetin-*O*-rhamnoside (226 μg/g dw) was the main compound found in the ethyl acetate extracts. The aqueous extracts presented higher amounts of flavan-3-ols (496 μg/g dw), while the ethyl acetate extract presented higher flavonols (550 μg/g dw) content. The extracts demonstrated high antioxidant activity. The antimicrobial activity of the extracts was only against Gram-positive bacteria, but some antifungal activity was also registered. For the first time, the antiproliferative activities associated with *C. villosus* extracts were reported [9].

The aqueous extracts of *C. scoparius* were studied for their phytotoxic potential in two agricultural weed species [42]. The study demonstrated that the phytotoxic effects of the *Cytisus* aqueous complex extracts were greater than isolated phenolic compounds, perhaps due to synergistic effects. The final conclusion was that the phytotoxic activity of the *C. scoparius* aqueous extracts is a complex result from the combined action of the polyphenols components and the complex interactions with other constituents like proteins, carbohydrates, and lipids.

*Cytisus multiflorus*, *C. scoparius*, and *C. striatus* were evaluated for their total phenolic content and antioxidant activity by several in vitro methods (DPPH scavenging activity, reducing power, β-Carotene bleaching inhibition, TBARS inhibition) [11]. *C. scoparius* demonstrated the highest content in total phenolic compounds, followed by *C. striatus* and *C. multiflorus*, with values of 427.10 mg GAE/g extract, 389.04 mg GAE/g extract, and 313.87 mg GAE/g extract, respectively. The authors reported that these species are widely used in folk medicine, and *C. multiflorus* are indicated for the prevention and treatment of diabetes, hypertension, hypocholesterolemia, headache and migraine, heart failure, rheumatoid arthritis, sores and cutaneous eruptions, acne, and as anti-inflammatory. *C. scoparius* are indicated for the prevention and treatment of metabolic illness, gout and rheumatic disorders, heart failure and tachycardia, joint and muscles pain, renal problems, skin inflammations, anti-hemorrhagic, cholagogue, diuretic, and vasopressor, among others. *C. striatus* are also traditionally indicated for the prevention and treatment of gout and rheumatic illnesses, hypotension, heart failure, anti-inflammatory, cardiotonic, cholagogue, diuretic, depurative, and vasopressor. The experimental results obtained by Pinela et al. [11] gave scientific support to the uses of these plants in traditional medicine as antioxidant with anti-inflammatory properties.

Only the flowers of *C. multiflorus* were evaluated for their polyphenol profile [25]. The *C*. *multiflorus* did not present phenolic acids and derivatives. The identified polyphenol compounds belong mainly to the groups of flavonols and flavones, represented by quercetin derivatives and chrysin derivatives, respectively. Similar results were published by [13] for the ethanol extracts of flowers of *C*. *multiflorus*. The most abundant polyphenol compound found in the extract were chrysin derivatives (23.4 mg/g dw) that accounted for more than 50% of the total polyphenol content (41.8 mg/g dw). The antioxidant and cells protective effects of isolated ethanolic extracts of *C. multiflorus* were investigated and compared to the activities of the main individual polyphenolic constituents (chrysin, apigenin, quercetin, and luteolin) [45]. The authors suggested that the polyphenol chrysin (72.8 mg/g extract) is the main constituent responsible for the high antioxidant activity of the extracts, as it accounted for 56% of the total polyphenolic content (130.3 mg/g extract). In contrast, the cytoprotective action of the extracts could not be related to the major polyphenol compounds.

The acetone extracts of aerial parts of Algerian *C. triflorus* show antioxidant, antibacterial, and anti-inflammatory effects, as well as wound healing properties [46]. The scientific results supported and justified the traditional use of this plant. Moreover, according to the authors, further researches are necessary to identify the phytochemicals which are responsible for these biological activities. The antioxidant activities of another *Cytisus* specie from Algeria, *C. monspessulanus*, were studied [47]. According to the authors, this is the first study reporting the total phenolic and flavonoid contents for *C. monspessulanus* extracts. It was noted that the extracts had important higher amounts of flavonols and flavones (20.4 mg/g dw) than flavanones and di-hydroflavonols (5.5 mg/g dw). Moreover, *C. monspessulanus* extracts had the highest content of flavonols and flavones compared to the other seven plant extract in the study, namely *Anthemis arvensis* L., *Haloxylon scoparium* Pomel, *Arbutus unedo* L., *Artemisia campestris* L., *Juniperus phoenicea* L., *Thymus algeriensis* Boiss et Reut, *Zizyphus lotus* L. (Desf.), and *C. villosus* Pourr. The hydroethanolic extract of the aerial part of this specie were studied by Larit et al. [48], and the major identified polyphenolic compounds were ginestein and chrysin.

As final remarks, we can conclude that extracts of *Cytisus* species are rich in polyphenolic compounds, especially chrysin and quercetin and its derivatives, and with powerful antioxidant, antimicrobial, etc., biological activities (Section 4).

Table 2 summarizes the phenolic composition of some species of the genus *Cytisus* that are reported in the bibliography. It is noticeable that the amount of phenolic compounds reported depends on factors such as the species of *Cytisus* used for extraction, the age and growth conditions of the plants, the part of the plant used (branches, leaves, flowers or others), the applied technology of extraction, the solvent (water, ethanol, ethyl acetate, etc.), and other conditions used for phenolic recovery and isolation (time, temperature, solid–liquid ratio, etc.). In this sense, differences can be observed in the results reported by different authors, in addition to the units used to express the results.

## 4. Emerging Applications of *Cytisus* Bioproducts

### 4.1. Potential Food and Food-Related Applications

The use of plant-based substitutes for meat, dairy, and others products of animal origin is well described in the literature. Its use was boosted due to an increase in vegan and/or vegetarian diets, environmental concerns, desire for sustainability, as well as an FAO recommendation for a healthier and sustainable and healthier diet, among other causes [49].

As mentioned in previous sections, plant and plant by-products have compounds in their composition that have a significant positive impact on human health, including polysaccharides, fibers, proteins, carotenoids, polyphenols, and sterols, among others. *Cytisus* plants have a rich composition of bioactive flavonoids and phenolic acids.

When discussing the possible applications of plant-based products in the food industry, there are several categories of food consumption products including proteins, whole grains, flour, oil, “milk”, sweeteners, and food additives (natural flavors and aromas, colorants, preservatives, etc.). Their incorporation into new food products or increasing the functionality of existing ones (functional foods) and in functionalized food packaging or films has also been explored [50,51,52,53].

As described by Zang et al. [54], natural food additives have drawn attention due to its green safety, health, and non-toxicity, this being an emerging field in the food industry.

Several species from the *Cytisus* genus have already been characterized in terms of their chemical and biological potential, as can be seen in Table 2 and Table 3, respectively. The phytochemical compounds detected in each species confers unique characteristics, with potential application in the food industry. As an example, *Cytisus* species, like *C. multiflorus*, *C. villosus*, and *C. scoparius*, which have in their composition compounds known for their antioxidant and anti-inflammatory properties, like gallic, protocatechuic, chlorogenic and caffeic acids, chrysin, rutin, kaempferol, and quercetin, can be incorporated into familiar foods like cookies and snacks to enhance their functional value. This addition of bioactive compounds can contribute to the prevention of several non-communicable diseases like diabetes, cardiovascular diseases, and even neurodegenerative diseases like Alzheimer’s [55,56,57].

Food preservatives are substances used to extend the shelf life of certain food products by preventing oxidation and avoid food degradation by microorganisms [54]. As reported previously, *Cytisus* extracts, independently of the species, have antioxidant and antimicrobial activities, making them an optimal source of food preservatives.

As described by Sahraee et al. [58], food deterioration can significantly alter the organoleptic qualities of food, therefore the food industry has been searching for ways to prevent this deterioration. For the past few years, synthetic antioxidants have been incorporated into food products and food packaging, although due to their toxicity, natural antioxidants have been sought, particularly plant-based ones [59]. Due to the rich composition in phenolic compounds of the *Cytisus* plants (Table 2), their extracts confer interesting antioxidant properties, which in combination with their antimicrobial characteristics makes them ideal for incorporation into edible films, for example. Therefore, plant-based products can be used as food additives, preservatives, or even food packing without being a hazard to the consumer, due to the low toxicity of most of the plant-based molecules [60].

The knowledge gathered in recent years regarding the beneficial effect of compounds present in the *Cytisus* genus opens the door to a widely vast array of applications for food and food-related applications, like safer and more efficient food additives, healthier diets to prevent non-communicable diseases (like cancer, diabetes, or cardiovascular diseases), and the improvement of food packaging materials, increasing their function, and reducing their carbon footprint by diminishing the use of synthetic alternatives.

### 4.2. Potential Health Applications

Phytochemicals have played an imperative role from ancient to recent times in the prevention and treatment of many illnesses with extensive effects, such as antioxidants which are directly linked with reduced risks of cancer, cardiovascular disease, diabetes, infectious diseases, and other disorders associated with age [61,62]. The benefit of many natural products, which have been part of the human diet for several thousand years, is that the human body has adjusted to them, which can lower the risk of damaging side effects [26].

Some *Cytisus* plants growing spontaneously in thermos-Mediterranean conditions and are widely used in local folk medicine and occasionally as condiments [11,13]. As reported before, for a long time, the extracts of these plants have been used for therapeutic purposes due to their hypnotic and sedative, diuretic, anti-diabetic, hepatoprotective, antioxidant, anti-inflammatory, antiparasitic, lithotriptic, cardiotonic, hypotensor, cathartic, and emetic properties.

**Figure 3:**
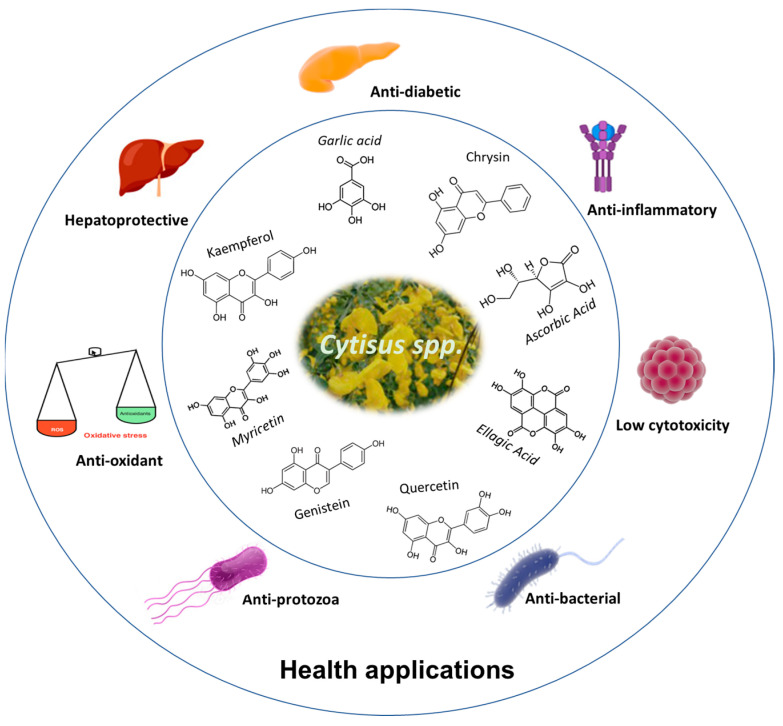
Potential health applications of *Cytisus* polyphenolic extracts.

The therapeutic properties of *Cytisus* spp. are mainly associated with its high concentration of polyphenolic compounds, as reported in the previous sections. The scheme in Figure 3 presents the main therapeutic actions reported so far for products derived from plants of the genus *Cytisus*.

#### 4.2.1. Antioxidant Activity

Antioxidant activity is one of the most studied biological properties of natural products, particularly medicinal and food plants.

Numerous studies have shown that the antioxidant action of plants could be related with protection against human diseases related to oxidative stress. In this regard, flavonoids and other polyphenol compounds have gained the most attention. The genus *Cytisus* contains the main components of phenolic acids and flavonoids with great antioxidant capacity [11,23,26,48]. In fact, *Cytisus* plants appear as an alternative to conventional plants as they have excellent antioxidant properties. This “opens the door” to providing added value from this bioresource to food and therapeutic applications, as a functional food, food supplement, or food additive (natural preservative).

Sundararajan et al. [24] reported that the extracts of *C. scoparius* aerial part (at the dose 104.0 µg/mL) present 50% protection against lipid peroxidation induced by Fe^2+^/ascorbate system in rat liver microsomal preparation. In addition, a decrease in the generation of the hydroxyl radical was visible with an IC_50_ value of 27.0 µg/mL for the *Cytisus* extracts when compared to the standard vitamin E (32.5 μg/mL).

Likewise, Raja et al. [63] have observed that the same plant extracts protect the liver from oxidative stress induced by carbon tetrachloride (CCl_4_) in rats at a dose of 250 and 500 mg/kg for 7 days. *C. scoparius* extracts also have anti-stress and moderate anxiolytic activity in rats at 125 and 250 mg/kg for 21 days. This effect may be due, in part, to its antioxidant effect [64]. In addition, González et al. [12] showed that these plant extracts present topical applications for skin protection against oxidative damage.

On the other hand, the antioxidant capacity of the *C. villosus* extracts and its isolated phenolics (genistein, chrysin, chrysin-7-*O-β-D*-glucopyranoside, and 2″-*O-α-L*-rhamnosylorientin) was measured in hepatocellular carcinoma cells (HepG2 cells) [26]. Results showed inhibition of intracellular oxidative stress (36% inhibition of ROS generation at 250 µg/mL). Concomitantly, the purified and isolated compounds were not effective, except for 2″-*O-α-L*-rhamnosylorientin from *n*-BuOH extract which presented 28% of ROS inhibition at 250 µg/mL. These results demonstrate the synergistic effect between natural compounds, in addition to the fact that the same phenomenon can occur between natural and synthetic compounds if administered at the same time.

By comparing the antioxidant activity of *Cytisus* extracts with other plants used in food and medicine, it is possible to perceive that the broom flower has similar or perhaps superior abilities to block and inhibit free radicals. For example, Ali-Jafri et al. [65] studied the in vitro antioxidant activity of different parts of traditional medicinal plants rich in polyphenols, and its results showed that 45 plant extracts (among them Thymus, Salvia, Melissa, *Veronica officinalis*, Mentha, etc.) have radical scavenging activity (IC_50_ values between 25–130 µg/mL for DPPH radical). In another study, Martins et al. [66] reported the in vitro antioxidant properties of edible wild greens in the traditional diet of the Iberian Peninsula (asparagus, white bryony, and black bryony). Their data showed DPPH radical scavenging effects with IC_50_ values of 423, 640, and 203 µg /mL for asparagus, white bryony, and black bryony, respectively. Comparing with *Cytisus* extracts, we observed that the antioxidant activity is similar (1.5 to 792 µg/mL for DPPH method) with these widely used plants. Furthermore, it is important to mention that the biological activity of the extracts cannot be literally compared with other studies because it depends on the antioxidant method (type of radical, reducing power, etc.), the species of *Cytisus*, the part of the plant used, and the extraction method used.

#### 4.2.2. Cytotoxicity and Antiproliferative Activity

Toxicological studies are very important for both synthetic and natural products before they can be administered and/or consumed by humans and animals. These types of tests are crucial to assess the safety of compounds and study the appropriate dose to obtain therapeutic effects. For this purpose, pre-clinical toxicity studies are essential, and many categories of test can be conducted, such as acute, subacute and chronic toxicity, cytotoxicity, teratogenicity, genotoxicity, etc.

To the best of our knowledge, only three studies have reported the cytotoxic activity of *Cytisus* spp. extracts (Table 3). The cytotoxicity of extracts and isolated compounds from *C. villosus* was determined against four human cancer cell lines (SK-MEL, KB, BT-549, and SKOV-3) and two non-cancerous kidney cell lines (LLC-PK and Vero). The tested extract/molecules were not active against any cell lines used in this study at 100 μM [26]. In the second study, the authors reported that the in vitro cytotoxicity of *C. scoparius* methanolic extract showed the IC_50_ value of 4.5 μg/mL on human cervical cancer cells (HeLa), which shows that the isolated compound genistein is cytotoxic [67]. The results of the cell cycle assay revealed the cell arrest at a sub-G phase, affirming the apoptotic effect of isolated genistein. Moreover, the human topoisomerase II test proved that it is a considerable human topoisomerase II inhibitor at 10 μg/mL [67]. In the third study, the authors revealed the cytotoxic/antiproliferative potential of *C. villosus* aqueous and ethyl acetate extracts on three human cancer cell lines of breast (T47D, MCF7) and colon (HCT-116) cancers. The results showed that the ethyl acetate extract exhibited higher inhibitory activity (IC_50_ values of 1.57 ± 0.06 mg/mL, 2.2 ± 0.1 mg/mL, and 3.2 ± 0.2 mg/mL for T47D, MCF-7, and HCT-116 cell lines, respectively) [9].

Due to the scarcity of studies on the cytotoxic potential of extracts or isolated molecules obtained from *Cytisus* spp., more work should be undertaken to validate their use in food and therapeutic applications, taking into account the doses tested.

#### 4.2.3. Anti-Inflammatory and Antidiabetic Activity

The genus *Cytisus* has been used for centuries and is reported to have anti-inflammatory properties. Ait-KaciAourahoun et al. [68] showed that the hydroalcoholic leaves extract from *C. triflorus* at a dose of 200 and 400 mg/kg body weight may be considered as a good anti-inflammatory agent in carrageenan-induced edema using a rat model. Likewise, extracts of *C. villosus* showed weak inhibition of iNOS with IC_50_ values of 48 µg/mL for EtOAc extracts to 90 µg/mL for *n*-BuOH in mouse macrophages (RAW 264 cells) [26]. These results encourage further investigation of the phytochemical compounds of *Cytisus* spp. regarding its anti-inflammatory potential and other biological actions.

Type 2 diabetes is one of the non-communicable diseases that is becoming a “pandemic” in the world. Traditional medicine has used herbs for the treatment of diabetes, either independently or in combination with food. Thus, scientific investigation on traditional herbal medicines for diabetes can provide valuable evidence for the development of alternative drugs and strategies, as shown by various studies carried out on plants from North Africa and Central Asia, including those of the genus *Cytisus* [61,69,70,71,72]. Recent studies have reported the effect of natural extracts rich in polyphenolics in the prevention and treatment of type 2 diabetes [73]. Some of these studies have confirmed the antidiabetic efficacy (antihyperglycemic activity) of extracts from various species of Fabaceae. Although there are no studies on the action of extracted compounds from *Cytisus* species, considering the composition in polyphenolics, they seem to have antidiabetic potential and more studies should be conducted both in vitro and in vivo.

#### 4.2.4. Hepatoprotective Activity

Raja et al. [63] showed that pretreatment of Wistar albino rats with *C. scoparius* extract (250 and 500 mg/kg) significantly affects liver injury. The levels of antioxidant enzymes like superoxide dismutase (SOD), catalase (CAT), reduced glutathione (GSH), glutathione peroxidase (GPx), glutathione-s-transferase (GST), and glutathione reductase (GRD) parameters were considerably increased by *C. scoparius* extract treatment in a rat model of carbon tetrachloride (CCl_4_) hepatic toxicity. Moreover, the capacity of the extract at the dose of 500 mg/kg was comparable to that of the standard drug, silymarin (25 mg/kg).

In the same way, CCl_4_ induced liver injury in rabbits, and *Cytisus* extracts orally in a dose of 400 mg/kg/day for 14 days reduced levels of hepatic enzymes like alanine transaminase (ALT) and aspartate transaminase (AST), alkaline phosphatase (ALP), and total bilirubin, showing preventive liver damage and improving biological functions of liver [74].

#### 4.2.5. Antimicrobial and Antiprotozoal Activities

Antibiotic resistance is one of the most critical situations facing humanity. Therefore, due to the increase in antibiotic resistance, it is very important to identify new and innovative antimicrobial agents. As mentioned previously, plants contain many phytochemicals that can be used for therapeutic purposes and generally have low toxicity [75,76,77].

The antibacterial activity of *C. villosus* extract was tested against *Escherichia coli*, *Staphylococcus aureus*, *S. epidermidis*, *Bacillus subtilis*, *Pseudomonas aeruginosa*, and *Mycobacterium intracellular* in comparison with standard antibiotics. However, the result was not significant against *E. coli* and *P. aeruginosa* [9,26]. Bouziane et al. [9] reported the selective action of *Cytisus* extracts and potent antimicrobial actions against the Gram-positive bacterium *S. epidermidis* (IC_50_ of 186 ± 9 μg/mL for aqueous extracts and 92 ± 3 μg/mL for ethyl acetate extracts). Likewise, the effect of the *Cytisus* extract against four fungi including *Aspergillus fumigatus*, *Mucor* specie, *Aspergillus niger* and *Fusarium solani* was explored, without finding significant results in its growth. Larit et al. [26] also evaluated the antifungal activity of this extract against a pathogenic fungus associated with opportunistic infections, including *Candida albicans*, *C. glabrata*, *C. krusei*, *A. fumigatus*, and *Cryptococcus neoformans*, and no relevant results were obtained. On the other hand, the same extracts presented an antitrypanosomal effect against Trypanosoma brucei at tested concentrations (7 to 19.48 µg/mL) [26].

Yahiaoui et al. [46] tested the antibacterial activity of *C. triflorus* extracts by microdilution method against *E. coli*, *S. aureus*, *B. subtilis* and *P. aeruginosa.* Their study demonstrated that all extracts were active against the tested bacteria, but more effective against Gram-positive ones. Benabderrahmane et al. [44] also suggested that *C. triflorus* polyphenols and flavonoids are responsible for its antioxidant capacity and antimicrobial properties against Gram-positive bacteria *S. aureus*.

In another study, Lores et al. [8] reported that the *C. scoparius* extracts obtained by PLE with ethyl lactate decreased the minimum inhibitory concentrations (MICs) from 3% to 2% for Gram-positive species (*S. aureus* and *Bacillus* spp.), demonstrating that the polyphenol-rich extract exerted a positive antibacterial activity. For a Gram-negative bacterium (*E. coli*) an increase in the MIC from 3% to 4% when using the extract was observed. On the other hand, experiments with the fungus *C. albicans* showed no anti-fungal activity for the *C. scoparius* extract.

In general, antimicrobial activity studies show that phytochemicals from different *Cytisus* extracts can confer some type of antibacterial action against various strains, and do not show antifungal activity.

The growing demand for new drugs with antibiotic effects has led to the study and adoption of new strategies to combat multidrug-resistant bacteria. Abreu et al. [78] tested 29 plant species as strategies to deal with methicillin-resistant *S. aureus* (MRSA). Interestingly, *C. striatus* extract presented antibacterial activity and acted as an antibiotic adjuvant, potentiating the effect of ciprofloxacin and erythromycin (commercial antibiotics) against MRSA strains. This study revealed the potential of *C. striatus* extracts for therapeutic applications in the control of infections caused by microorganisms’ resistant to antibiotics.

**Table 3:** Potential health applications of *Cytisus* plant extracts.

Activity	Assay Model	Extracts	Effect/Methodology	Reference
Antioxidant	In vitro	- Aerial part C. scoparius - Ethanol-water (70:30 *v*/*v*) extracts	*IC_50_ activity:* 1.5 µg/mL for DPPH radicals; 116 µg/mL nitric oxide; 4.7 µg/mL superoxide anion; 27.0 μg/mL hydroxyl radical; potent antioxidant reducing power	[24]
	In vitro	- Aerial part *C. triflorus* - Hydroalcoholic extracts EtOH:H_2_O (80:20) extracts	DPPH radicals scavenging (IC_50_): 19.17 and 77.8 µg/mL for leaf and stem, respectively	[68]
	In vitro	- Aerial part *C. villosus* - Hydroalcoholic extracts partitioned with CHCl_3_, EtOAc and n-BuOH	DPPH radicals scavenging (IC_50_): 459 µg/mL CHCl_3_ extract; 425 µg/mL EtOAc extracts; 164 µg/mL for n-BuOH	[26]
	In vitro	- Aerial part *C. triflorus* - Acetone extract	DPPH radicals scavenging (IC_50_): 792 ± 10 µg/mL	[46]
	In vitro	- *C. villosus* aerial parts - Ethyl acetate and aqueous extracts	DPPH radicals scavenging (IC_50_): 59 ± 2 µg/mL for aqueous extracts and 31 ± 2 for ethyl acetate extracts	[9]
	In vitro	- Aerial parts *C. scoparius* - *Ethyl lactate extracts* - *Methanol extracts*	Greater scavenging of DPPH radicals by ethyl lactate:water (65:35 (*v*:*v*)) extracts (approx. 6 mM Trolox/g plant)	[8]
	Rat liver microsomal preparation	- Aerial part *C. scoparius* - Ethanol:water (70:30 *v*/*v*) extracts	Protection in lipid peroxidation at 104 µg/mL	[24]
	Rat liver	- Aerial part *C. scoparius* - Ethanol:water (70:30 *v*/*v*) extracts	Protects liver from oxidative stress at 250 and 500 mg/kg for 7 days	[63]
	Rat	- Aerial part *C. scoparius* - 60% methanolic extracts	Anti-stress at 125 and 250 mg/kg for 21 days	[64]
	Human epidermis	- Branches *C. scoparius* - Acetone:water and acidified ethanolic extracts	Skin protection against oxidative damage. In vitro assays with the reconstituted human epidermis, topical use at 1%	[12]
	Human hepatocytes	- Aerial part *C. villosus* - Hydroalcoholic extracts partitioned with CHCl_3_, EtOAc and n-BuOH	Inhibition of intracellular oxidative stress in HepG2 cells at 250 µg/mL	[26]
Cytotoxicity and Antiproliferative activity	Human cancer cells	- Whole *C. scoparius* - Methanolic extracts	Human cervical cancer cell line (HeLa); MTT assay, cell cycle analysis, and topoisomerase II inhibitory activity IC_50_ value of 4.5 μg/mL; cell arrest at a sub-G phase; promote apoptosis; excellent human topoisomerase II inhibitor at 10 μg/mL	[67]
	Human cancer cells	- Aerial part *C. villosus* - Hydroalcoholic extracts partitioned with CHCl_3_, EtOAc and n-BuOH	No toxicity at 100 µM against human cancer cell lines (SK-MEL, KB, BT-549, and SKOV-3) and two noncancerous kidney cell lines (LLC-PK1 and Vero)	[26]
	Human cancer cells	- *C. villosus* aerial parts - Ethyl acetate and aqueous extracts	Growth inhibition of the three different cell lines of breast (T47D, MCF7) and colon (HCT-116) cancers Ethyl acetate extract exhibited higher activity (IC_50_ values of 1.57 ± 0.06 mg/mL, 2.2 ± 0.1 mg/mL, and 3.2 ± 0.2 mg/mL for T47D, MCF7, and HCT-116 cells)	[9]
Anti-Inflammatory	Rat	- Leaves *C. triflorus* - Hydroalcoholic EtOH-in Eth-H_2_O (80:20) extracts	Anti-inflammatory agent at 200 and 400 mg/kg body weight	[68]
	Mousemacrophages	- Aerial part *C. villosus* - Hydroalcoholic extracts partitioned with EtOAc and n-BuOH	EtOAc and n-ButOH extracts showed inhibition of iNOS with IC50 of 48 and 90 µg/mL, respectively.	[26]
Hepatoprotective	Rats	- Aerial part *C. scoparius* - Ethanol:water (70:30 *v*:*v*) extracts	Increase levels of SOD, GS, and GRD parameters in carbon tetrachloride treated wistar albino rats at 250 and 500 mg/kg/day for 7 days	[63]
	Rabbits	- Whole *C. roseum* - Aqueous extract	Oral administration of extracts at 400 mg/kg/day for 14 days. Reduction in ALP, ALT, AST levels and total bilirubin in carbon tetrachloride treated rabbits	[74]
Antimicrobial and Antiprotozoal	*Staphylococcus aureus, Bacillus subtilis, Pasture llamultacida*	- Whole *C. roseum* - Aqueous and n-BuOH extracts	Anti-bacterial activity by disc diffusion method (30 µg/disc): 13.8 ± 0.18 (maximum concentration)	[74]
	*Trypanosoma bruces*	- Aerial part *C. villosus* - Hydroalcoholic extracts partitioned with EtOAc and n-BuOH	Antitrypanosomal activity (IC_50_): 19.48 µg/mL for EtOH extract; 7.99 µg/mL for n-BuOH	[26]
	*Staphylococcus aureus, Bacillus subtilis, Pseud* *omonas aeruginosa, Escherichia coli*	- Aerial part *C. triflorus* - Acetone extract	Minimal inhibitory concentration (MIC) assays *S. aureus*: 0.26 ± 0.09; *B. subtilis*: 0.42 ± 0.18; *P. aeruginosa*: 1.67 ± 0.72; *E. coli*: 1.04 ± 0.36 (values in mg/mL)	[46]
	*Staphylococcus aureus, Bacillus* spp.*, Escherichia coli, Candida albicans*	- Aerial parts *C. scoparius* - Ethyl lactate:water (65:35 *v*:*v*) extracts	MIC assayExtract exerted a positive antibacterial action	[8]
	*Staphylococcus aureus*	- *C. striatus*	Act as antibiotic adjuvants	[78]
	*Staphylococcus epidermidis, Escherichia coli, P* *seudomonas aeruginosa, Candida glabrata*	- *C. villosus* aerial parts - Ethyl acetate and aqueous extracts	Selective and potent antimicrobial activities against the Gram-positive bacterium (*S. epidermidis*) IC_50_ of 186 ± 9 μg/mL for aqueous extracts and 92 ± 3 μg/mL for ethyl acetate	[9]
Wound healing	Rats	- Aerial part *C. triflorus* - Acetone extract	Excision wound healing model for 14 days; results indicate re-epithelialization and good healing potential using 5% of the extract	[46]

SK-MEL: Human malignant melanoma; KB: Human epidermoid carcinoma; BT-549: Human ductal carcinoma; SK-OV-3: Human ovary carcinoma; LLC-PK-1: Pig kidney epithelial cells; Vero: African green monkey kidney cell line.

## 5. Conclusions and Perspectives

The interest for “Clean label” products, such as functional foods, nutraceuticals, biocosmetics, and natural drugs (pharmaceuticals), is motivated by imperative factors such as health and sustainability. This phenomenon is expressed in the consumer’s interest in natural, functional, and health-promoting products that are plant-based. The processing of undervalued plant by-products by innovative and green technologies can satisfy this growing interest, in addition to minimizing the generation of biowastes, reducing energy consumption, and increasing the sustainability of the remaining industries. Therefore, the valorization of underexplored plants and the reuse of agricultural residues, in this case *Cytisus* plants, can be a viable option to obtain high-value products (e.g., bioactive polyphenols) for the food, cosmetic, and pharmaceutical industries, such as antioxidants, dyes, flavorings, antimicrobial agents, or isolated drugs with highlighted functional and therapeutic potential. On the other hand, future detailed technoeconomic, environmental, and social assessments of bioprocessing of underexploited plant materials and design of new or improved plant-based products are required.

## Figures and Tables

**Table 2:** Phenolic composition of some *Cytisus* species reported in the literature.

Specie	Polyphenolic Compounds	Reference
*C. monspessulanus*	Flavonols and flavones (20.4), flavanones and di-hydroflavonols (5.5) total phenolic content (66.2) (values in mg/g dry matter)	[47]
*C. multiflorus*	Gallic acid (1.6), vanillic acid (1.2–4.3), caffeic acid (1.0–2.0), chlorogenic acid (2.2–3.4), syringic acid (0.7–2.9), *p*-coumaric acid (1.3–6.9), ferulic acid (1.8–25.4), ellagic acid (5.4–16.1), quercetin (3.0–37.1) (values in mg/g dry matter)	[40]
*C. multiflorus*	Quercetin derivative (4.1), luteolin derivative (4.0), kaempferol derivative (8.4), chrysin derivative (35.1) (values in mg/g dry matter)	[25]
*C. multiflorus*	Orientin (0.8), rutin (4.5), apigenin-7-*O*-glucoside (0.8) and chrysin (0.5), luteolin derivatives (6.8) (values in mg/g dry matter)	[13]
*C. multiflorus*	Chrysin derivative (72.8), luteolin derivative (23.4), apigenine derivative (20.0), quercetin derivative (14.1) (values in mg/g extract)	[45]
*C. multiflorus* *C. scoparius* *C. striatus*	Total phenolic content in mg GAE/g extract: *C. multiflorus* (313.87); *C*. *scoparius* (427.10); *C. striatus* (389.04) Total flavonol content in mg QE/g extract: *C. multiflorus* (72.64); *C*. *scoparius* (45.55); *C. striatus* (86.98)	[11]
*C. scoparius*	Protocatechuic acid (0.28–23.2), protocatechualdehyde (2.16–110), caffeic acid (0.70–22.3), orientin (1.10–220), kaempferol (31.9–309), rutin (1.34–552), isoquercetin (0.19–244), quercetin (0.70–219), apigenin (3.18–15.8), chrysin (0.36–172) (values in μg/g dry matter)	[8]
*C. scoparius*	Caffeic acid (0.7–2.9), cinnamic acid (0.2–0.7), vanillin (0.18–0.53), *p*-coumaric acid (1.2–6.1), ferulic acid (0.5), ellagic acid (0.9–2.1), quercetin (1.5), luteolin (1.2) (values in μg/mL)	[42]
*C. scoparius*	Gallic acid, protocatechuic acid, chlorogenic acid, caffeic acid, rutin, kaempferol, quercetin (compounds were only identified and not quantified)	[12]
*C. triflorus*	Chrysin plus derivatives (72.8), luteolin derivatives (23.4), apigenin plus derivatives (20.0), quercetin derivatives (14.1) (values in mg/g extract)	[46]
*C. triflorus*	Phenolics: 180.33 µg GAE/mg extract; Flavonoids: 16.78 µg QE/mg extract Individual compounds: 5-Hydroxy-7-*O*-glucosyl-flavone, 5-Hydroxy-7-*O*galactosyl-flavone, 5,7-Dihydroxy-flavone, 5,7,3′-Trihydroxy,4′-methoxy-flavone	[44]
*C. villosus*	Chrysin, chrysin derivative, genistein (compounds were only identified and not quantified)	[48]
*C. villosus*	Phenolics: 697 µg/g extract (ethyl acetate) and 712 µg/g extract (aqueous) Individual phenolics in μg/g dry matter: epigallocatechin derivatives (422.0 in water extracts; 110.5 in ethyl acetate extracts), catechin (36.1 only found in ethyl acetate extracts), apigenin derivatives (13.7 only found in water extracts), myricetin derivatives (141.0 in water extracts; 328.6 in ethyl acetate extracts), quercetin derivatives (38.7 in water extracts; 192.9 in ethyl acetate extracts), kaempferol derivatives (11.8 in water extracts; 27.4 in ethyl acetate extracts)	[9]

## Data Availability

Not applicable.
